# Effect of inflammation on association between cancer and coronary artery disease

**DOI:** 10.1186/s12872-023-03613-0

**Published:** 2024-01-24

**Authors:** Mingzhuang Sun, Shaoning Zhu, Yihao Wang, Yawei Zhao, Kaixin Yan, Xiaolong Li, Xueting Wang, Changjian He, Chunhua Ding, Yundai Chen, Zhijun Sun, Shunying Hu

**Affiliations:** 1 Department of Cardiology, Chinese PLA General Hospital, #28 Fuxing Rd, Beijing, 100853 China; 2 Cardiac Department, Aerospace Center Hospital, Peking University Aerospace School of Clinical Medicine, Beijing, China

**Keywords:** Cancer, Coronary artery Disease, SYNTAX score, Inflammation

## Abstract

**Background:**

Cancer and coronary artery disease (CAD) is reported to often co-exist in same individuals, however, whether cancer is directly associated with anatomical severity of CAD is rarely studied. The present study aimed to observe the relationship between newly diagnosed cancer and anatomical severity of CAD, moreover, to investigate effect of inflammation on the relationship of cancer with CAD.

**Methods:**

374 patients with newly diagnosed cancer who underwent coronary angiography (CAG) were enrolled. Through 1:3 propensity score matching (PSM) to cancer patients based on the age and gender among 51,106 non-cancer patients who underwent CAG, 1122 non-cancer patients were selected as control patients. Anatomical severity of CAD was assessed using SYNTAX score (SXscore) based on coronary angiographic image. SXscore ≤ 22 (highest quartile) was defined as SX-low, and SXscore > 22 as SX-high. The ratio of neutrophil to lymphocyte count (NLR) was used to describe inflammation level. Association between cancer and the anatomical severity of CAD was investigated using logistic regression.

**Results:**

Univariate logistic regression analysis showed a correlation between cancer and anatomical severity of CAD (OR: 1.419, 95% CI: 1.083–1.859; *P* = 0.011). Cancer was associated with increased risk of SX-high after adjusted for common risk factors of CAD (OR: 1.598, 95% CI: 1.172–2.179, *P* = 0.003). Significant association between cancer and SX-high was revealed among patients with high inflammation (OR: 1.656, 95% CI: 1.099–2.497, *P* = 0.016), but not among patients with low inflammation (OR: 1.530, 95% CI: 0.973–2.498, *P* = 0.089).

**Conclusions:**

Cancer was associated with severity of CAD, however, the association between the two diseases was significant among patients with high inflammation rather than among patients with low inflammation.

## Introduction

Cancer and cardiovascular disease are the two leading causes of death worldwide. The coexistence of cancer and cardiovascular disease is not uncommon [1, 2]. Cancer survivors are at an increased risk for cardiovascular disease [3]. Patients with CAD have a higher risk of cancer compared with the general population, according to the Second Manifestations of ARTerial disease (SMART) Study [4]. Inflammation is a common risk factor for CAD and cancer [5–7]. After adjusting the conventional risk factors for both diseases, recent research has shown the direct connection between atherosclerosis and cancer, which speculated that the link between the two diseases involves inflammation [8, 9].

Current research on the association between cancer and cardiovascular disease focuses more on anti-cancer therapy-related cardiotoxicity. However, whether there is a direct association between untreated cancer and CAD is getting more attention. A study in Japan showed that atherosclerotic cardiovascular disease might have a potential risk for cancer development [10]. Another study shows that myocardial infarction (MI) accelerates breast cancer outgrowth and cancer-specific mortality in mice and humans [11]. Newly diagnosed untreated cancer is also associated with CAD. According to a Canadian study, a new cancer diagnosis is independently associated with a significantly increased risk of cardiovascular death and nonfatal morbidity [12]. However, whether cancer is associated with the anatomical severity of CAD is rarely reported. To address this issue, we aimed to discover the association between newly untreated diagnosed cancer and the anatomical severity of CAD, and further investigate the effect of inflammation on the relationship of cancer with anatomical severity of CAD.

Inflammation is considered a common risk factor for both CAD and cancer [13]. Inflammation may lead to the development of AF, atherosclerosis, and cancer [14–16]. Various inflammatory biomarkers such as C-reactive protein, interleukin 6, and tumor necrosis factor α, et al. can reflect the level of inflammation [2]. NLR, the absolute neutrophil to lymphocyte ratio in peripheral blood, is a significant predictive inflammatory marker in various diseases [6, 17–19]. Compared with other inflammation biomarkers, NLR is a parameter readily available from routine blood cell counts [2]. NLR independently predicts atherosclerotic events and is a potential biomarker for residual inflammatory risk [7, 20]. Recent research has discovered that the severity and prognosis of numerous cardiovascular diseases are associated with NLR [21]. We adopted NLR to stratify the inflammatory state in order to further investigate the relationship between cancer and the anatomical severity of CAD under different inflammatory states.

## Methods

### Study patients

In Cardiac Department, the First Medical Centre, Chinese PLA General Hospital, Beijing, China, all information of inpatients was kept in the medical record system including coronary angiograms. Among 51,928 patients who underwent coronary angiography (CAG) (ICD-9-CM codes: 88.5, 88.55, 88.56, 88.57) between January 1, 2009, and December 31, 2020, there were 822 patients with a history of cancer including 374 newly diagnosed cancer patients without previous anti-cancer therapy until CAG. All the enrolled study patients who had CAD confirmed or suspected CAD had undergone CAG which was decided by his or her doctor. 374 newly diagnosed cancer patients had pathological cancer diagnosis. The enrolled cancer patients were transferred to cardiac department due to CAD or suspected CAD and had not received any anti-cancer treatments (radiotherapy and chemotherapy, targeted drug therapy, surgery, et al.) until CAG. Among the 51,106 non-cancer patients, 1122 patients were enrolled as control patients by 1:3 propensity score matching to the included cancer patients according to gender and age. All patients with infection, autoinflammatory diseases, previous percutaneous coronary intervention (PCI) or coronary artery bypass grafting (CABG) were excluded.

**Fig. 1:**
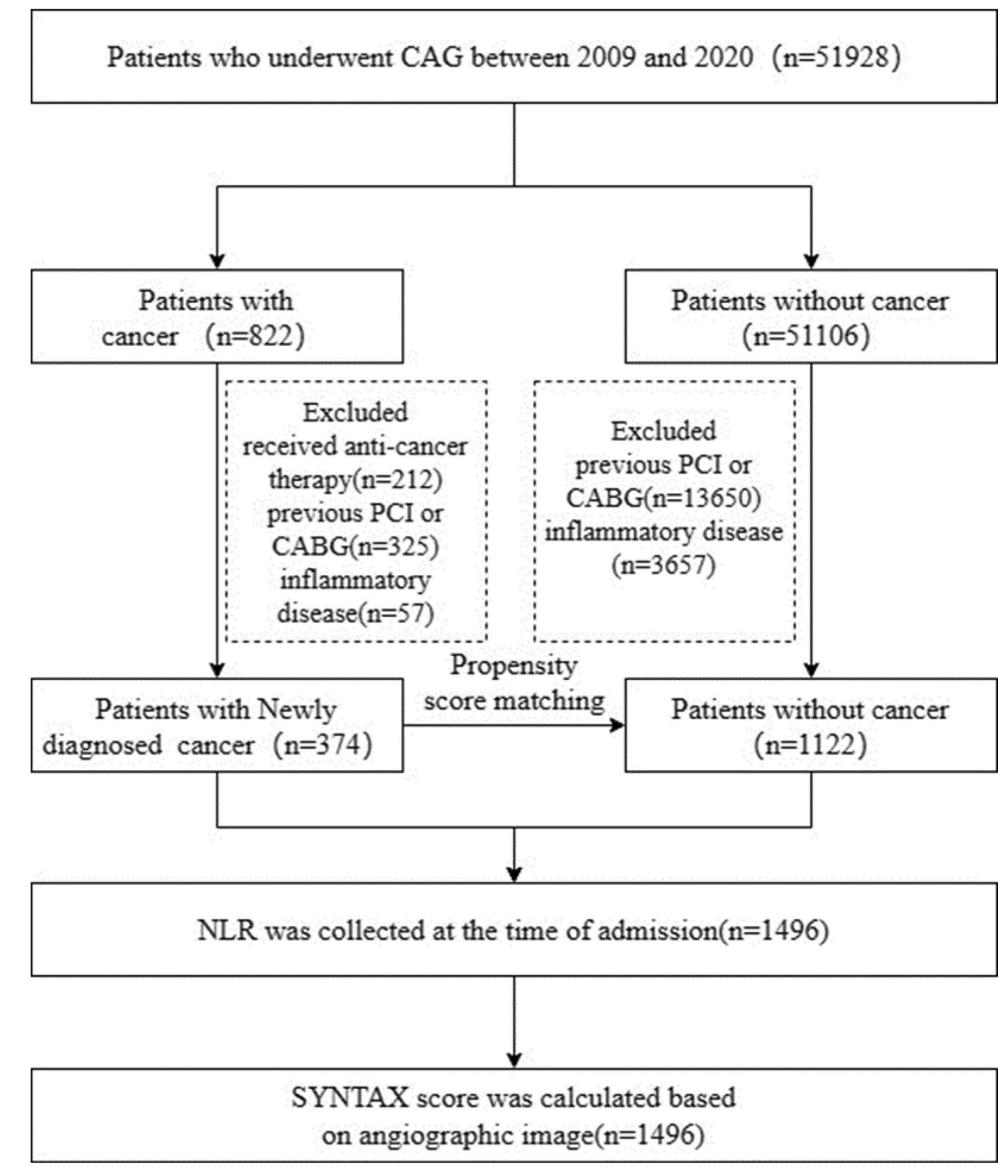
Flowchart of study patients

A flowchart of the study patient enrollment process was shown in Fig. 1. The study protocol was approved by the Institutional Review Board of Chinese PLA General Hospital (Ethical approval number: S2022-254-01).

### Assessment of anatomical severity of CAD based on CAG

The anatomical severity of CAD was evaluated using the SXscore algorithm (described in full elsewhere) [22]. Two blinded experienced interventional cardiologists computed all of the angiographic variables related to the SXscore calculation. When the SXscore of each patient differed between the two cardiologists, they would discuss the angiograms and reach a common SXscore. Final SXscores were calculated per patient and saved in a dedicated database. The SXscore of 21.5 was the highest quartile in the study. A low SXscore (SX-low) was defined as SXscore ≤ 22, and a high SXscore (SX-high) as SXscore > 22.

### Assessment of inflammatory status in patients

In order to assess the inflammatory status of study patients, we made use of NLR as the inflammatory marker. Complete blood cell counts, including the total white blood cells, neutrophils, and lymphocytes, were obtained from the medical record system tested at admission. NLR was calculated as neutrophil count divided by lymphocyte count. In the study, the median of NLR was 2.217, so we defined NLR grade as NLR-low (NLR ≤ 2.217) and NLR-high (NLR > 2.217).

### Statistical analysis

Descriptive statistics were presented as frequency and percentage rates for categorical variables, mean ± standard deviation (SD) and medians [interquartile range (IQR)] for continuous variables, according to the normality of the data. We assessed the normality of the data using the Skewness and Kurtosis normality tests. We used the Independent-Samples t-test to compare means between groups when variables were normally distributed. We assessed the relationship between cancer and CAD severity by using the logistic regression analysis, adjusting for common related risk factors, including age, gender, BMI, smoking, alcohol consumption, diabetes, hypertension, hyperlipidemia, family history of CAD and NLR. Two-sided P-value < 0.05 were considered statistically significant, and the odds ratio (OR) was presented with 95% confidence interval (CI). Statistical analysis was performed using SPSS 22.0 (SPSS Inc., IBM, Chicago, IL, USA).

## Results

### Patients’characteristics

**Fig. 2:**
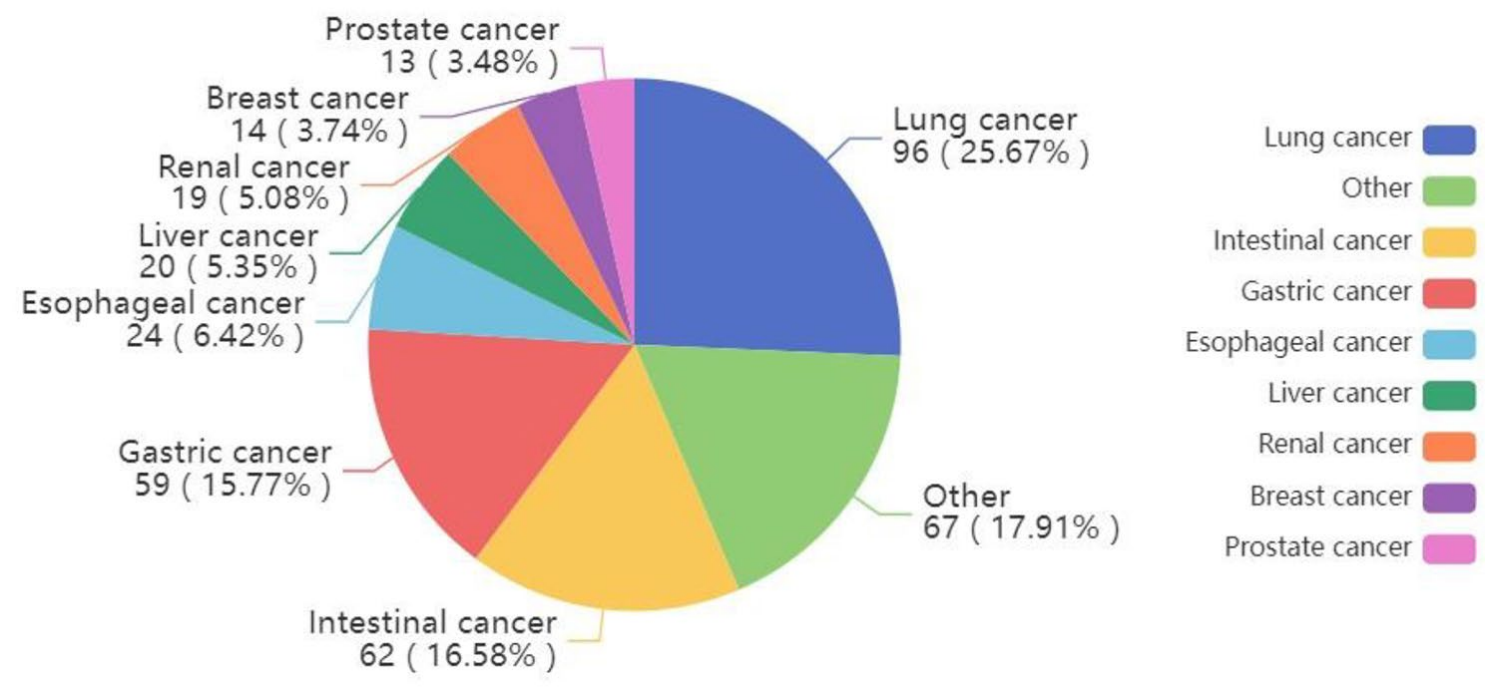
Proportion of various cancers. Among the 374 cancer cases, lung cancer accounted for 25.67% (96 cases), followed by intestinal cancer 16.58% (62 cases), gastric cancer 15.77% (59 cases), esophageal cancer 6.42% (24 cases), liver cancer 5.35% (20 cases), renal cancer 5.08% (19 cases), breast cancer 3.74% (14 cases), prostate cancer 3.48% (13 cases), and other cancers 17.91% (67 cases)

1496 patients, 1111 males and 385 females, with an average age of 65.7 ± 9.2 years, were included. There were 374 patients with cancer and 1122 patients without cancer. The median time from cancer diagnosis to patient undergoing CAG was 22.5 days. Among the 374 patients, 96 (25.67%) had lung cancer, 62 (16.58%) had intestinal cancer, 59 (15.77%) had gastric cancer, 24 (6.42%) had esophageal cancer, 20 (5.35%) had liver cancer, 19 (5.08%) had renal cell carcinoma, 14 (3.74%) had breast cancer, 13 (3.48%) had prostate cancer, and 67 (17.91%) had other cancers (Fig. 2).

**Fig. 3:**
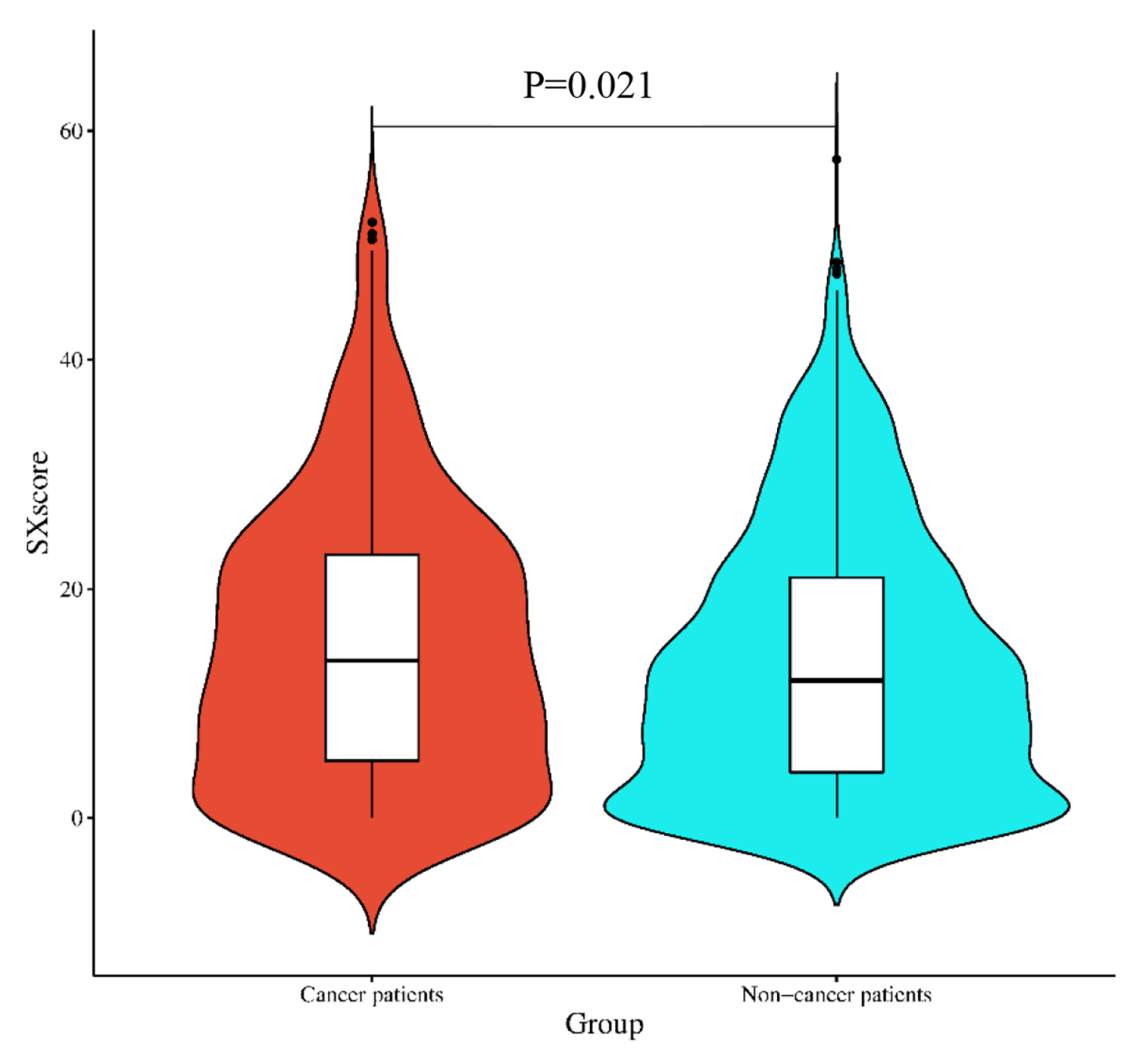
Distribution of SXscore in study Patients. Cancer patients showed higher SXscore than non-cancer patients when SXscore was considered as a continuous variable (*p* = 0.021)

**Fig. 4:**
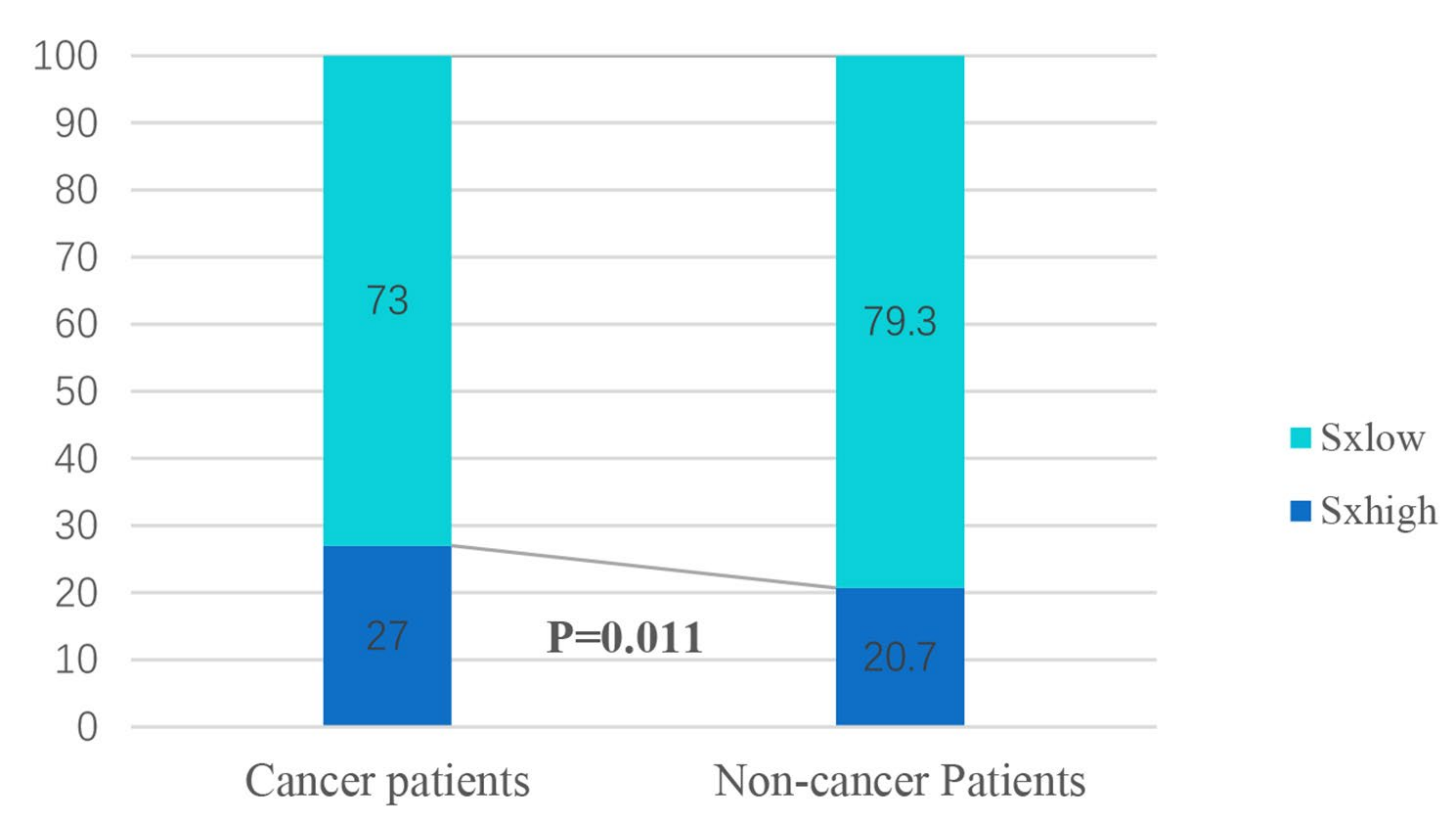
Distribution of SX-high and SX-low in study Patients. The proportion of SX-high among cancer patients was higher than that among Non-cancer patients when SXscore was considered as a categorical variable (27.0%vs. 20.7%, *p* = 0.011)

The upper quartile of the 1496 enrolled individuals had a SXscore of 21.5, the median was 12, and the Lower quartile was 4. Patients with cancer had significantly higher SXscore than those who did not (*P* = 0.021) (Fig. 3). Out of 1496 patients, 333 were classified as SX-high and 1163 as SX-low. The proportion of patients with SX-high among cancer patients was higher than those without cancer (27.0%vs. 20.7%, *P* = 0.011) (Fig. 4). 1466 patients in all, out of 1496 patients, had their NLR data taken. Patients with cancer had higher NLR values than those without cancer (2.513(IQR[1.586–2.976]) vs. 2.394(IQR[1.697–3.177]), *P* = 0.011). And when the median NLR (2.217) was used as the cutoff point (NLR-low 2.217, NLR-high > 2.217), the proportion of NLR-high patients in cancer patients was significantly higher than that in non-cancer patients (56.7% vs. 47.9%, *P* = 0.004) (Table 1).

**Table 1:** Clinical characteristics of study patients

Variables	cancer patients (N = 374)	Non-cancer patients(N = 1122)	*P* Value
Gender			
Male Female	282(75.4%) 92(24.6%)	829(73.9%) 293(26.1%)	0.562 ^a^
Age (yrs)	66.0(IQR[60.0–70.0])	66.0(IQR[59.0–73.0])	0.821 ^b^
BMI(Kg/M^2)	24.80(IQR[22.60–26.90])	25.10(IQR[23.30–27.20])	0.228 ^b^
Smoking history			
Never-Smoker Current-smoker Ex-smoker	190(50.8%) 169(45.2%) 15(4.0%)	585(52.1%) 419(37.3%) 118(10.5%)	< 0.001 ^a^
Drinking history			
Non-drinker Current-drinker Ex-drinker	218(58.3%) 125(33.4%) 31(8.3%)	709(63.2%) 361(32.4%) 52(4.6%)	0.019 ^a^
Family history of CAD	41(11.0%)	159(14.2%)	0.112 ^a^
Hypertension	220(58.8%)	663(59.1%)	0.927 ^a^
Diabetes Mellitus	130(34.8%)	345(30.7%)	0.149 ^a^
Hyperlipidemia	28(7.5%)	209(18.6%)	< 0.001 ^a^
NLR	2.513(IQR[1.586–2.976])	2.394(IQR[1.697–3.177])	0.011 ^b^
NLR classification			
NLR-high NLR-low	199(56.7%) 152(43.3%)	534(47.9%) 581(52.1%)	0.004 ^a^

Note: a: Categorical variables, using chi-square test; b: Continuous variables, non-normally distributed, using non-parametric tests Mann-Whitney U

Current-smoker, Current-drinker, Hyperlipidemia, SXscore and NLR are different between cancer and non-cancer patients (Table 1).

### Association between cancer and CAD in total patients

After adjusting for age, sex, BMI, smoking, drinking, diabetes, hypertension, hyperlipidemia, and family history of CAD, multivariate logistic regression analyses showed that the cancer was associated with SX-high. After adjusting for confounders, the risk of SX-high was 1.456 times higher in cancer patients than in non-cancer patients (95% CI: 1.097–1.934; *P* = 0.009) (Table 2).

**Table 2:** Multivariable logistic regression ORs and 95%CIs of cancer for SX-high

Model	OR	95%CI	*P* value
Model 1	1.419	1.083–1.859	0.011
Model 2	1.433	1.088–1.887	0.011
Model 3	1.456	1.097–1.934	0.009

Note: Model 1: unadjusted; Model 2: Age ^b^, gender ^a^ and body mass index ^b^ were adjusted; Model 3: on the basis of Model 2, adjust smoking history ^a^, drinking history ^a^, family history of CAD ^a^, hypertension ^a^, diabetes ^a^, hyperlipidemia ^a^;

a: categorical variables; b: continuous variables

### Association of cancer with CAD in patients with different inflammation level

Among patients with NLR-high, logistic regression analysis showed that cancer increased the risk of SX-high. After adjusting for age, gender, BMI, smoking, drinking, diabetes, hypertension, hyperlipidemia, and family history of CAD, the risk of SX-high in patients with cancer was 1.518 times higher than that in patients without cancer (95% CI: 1.097–1.934; *P* = 0.030) (Table 3).

**Table 3:** Multivariable logistic regression ORs and 95%CIs of cancer for SX-high in different inflammation level

Model	NLR-high group			NLR-low group		
	OR	95%CI	*P* value	OR	95%CI	*P* value
Model 1	1.481	1.032–2.124	0.033	1.289	0.853–1.948	0.228
Model 2	1.519	1.055–2.188	0.025	1.293	0.843–1.928	0.239
Model 3	1.518	1.042–2.212	0.030	1.390	0.891–2.168	0.147

Note: Model 1: unadjusted; Model 2: Age ^b^, gender ^a^ and body mass index ^b^ were adjusted; Model 3: on the basis of Model 2, adjust smoking history ^a^, drinking history ^a^, family history of CAD ^a^, hypertension ^a^, diabetes ^a^, hyperlipidemia ^a^;

a: categorical variables; b: continuous variables

Among the patients with NLR-low, OR of cancer for SX-high was 1.390 (95% CI: 0.891–2.168, *P* = 0.147). The results showed that cancer was not significantly associated with SX-high in patients with NLR-low (Table 3).

## Discussion

The present study demonstrated that newly diagnosed cancer was associated with the anatomical severity of CAD. Moreover, subgroup analysis indicated that the association was significant among patients with high inflammation but not significant among patients with low inflammation.

Advancements in early cancer diagnosis and therapy have significantly prolonged the life expectancy of cancer patients. With longer survival, new-onset CAD is frequently encountered in patients with cancer, affecting their subsequent morbidity, mortality, and healthcare costs [23]. In addition to cancer-related issues, studies have indicated that cardiovascular cause is the leading cause of death in cancer patients [24]. The association between cancer and cardiovascular disease has attracted considerable attention in recent years. However, these studies focus mainly on anti-cancer therapy-related cardiotoxicity [1, 25, 26], but rarely on the association between cancer and anatomical severity of CAD. This study showed that newly diagnosed untreated cancer was independently associated with the anatomical severity of CAD, and the association was more significant among patients with high inflammation than those with low inflammation. The results indicated that inflammation made a crucial difference between cancer and CAD.

The increased risk of CAD in patients with cancer is attributable to a combination of shared risk factors and common mechanisms. Cancer and CAD share common risk factors, such as obesity, hypertension, diabetes, hyperlipidemia, smoking, drinking, and so on [27–29]. In the present study, patients without anti-cancer therapy were chosen, and the multivariable regression model involved age, gender, smoking and drinking history, diabetes and hypertension history, and hyperlipidemia. The findings demonstrated that cancer was an independent risk factor for more severe CAD.

Recent evidence suggests that inflammation is the reciprocal pathophysiologic link between atherothrombosis and cancer [30]. Coronary artery disease is associated with increased levels of proinflammatory cytokines such as IL-1β, IL-6, interferon-γ (IFN-γ), and TNF-α [31]. Elevations in C-reactive protein and IL-6 have been associated with increased risk for cardiovascular events independent of cholesterol level [32]. Additionally, NLRP3 inflammasome activation and IL-1β have been shown to promote atherogenesis and arterial thrombosis in preclinical animal models [33]. Whether anti-inflammatory drugs can carry out targeted intervention on inflammation and positively impact the prognosis of patients with coronary artery disease has attracted increasing attention. The CANTOS (Canakinumab Anti-Inflammatory Thrombosis Outcomes Studies) shows that anti-inflammatory therapy with Canakinumab could significantly reduce the incidence of nonfatal myocardial infarction, nonfatal stroke, and cardiovascular death [34]. As another anti-inflammatory drug, Colchicine also plays a pivotal role in reducing the rate of cardiovascular events among patients with a recent myocardial infarction [35].

Inflammation also makes a difference in the development and progression of cancer [36]. Preclinical and clinical evidence demonstrates that cancer progression is determined by the tumor genetic landscape and complex interactions within the systemic host milieu and tumor microenvironment [37]. Inflammation is one of the essential elements in the microenvironment [37]. Inflammatory cytokines, such as interleukin (IL)-1, IL-6, IL-10, tumor necrosis factor-α, macrophage migration inhibitory factor, and transforming growth factor-β, are involved in tumor initiation and progression [14, 38]. An analysis of CANTOS suggests the possibility that anti-inflammatory therapy with canakinumab targeting the interleukin-1β innate immunity pathway could significantly reduce the incidence and mortality of lung cancer [39]. Recently, the presence of age-related clonal hematopoiesis, the so-called clonal hematopoiesis of indeterminate potential, has also been proposed as one of the vital pro-inflammatory drivers for a link between cancer and CAD [40]. A recent study reported that local coronary inflammation is associated with coronary events, which may work together with systemic inflammation to affect the severity of CAD [41]. It is also a potential mechanism that links inflammation in cancer patients to the severity of CAD.

The present study found no significant correlation between cancer and SX-high in the low inflammation group. In contrast, there was a significant correlation in the high inflammation group. Consequently, the association of cancer and SX-high may be related to a high inflammatory state. It was reasonable to infer that inflammation is essential to the link between cancer and CAD, and high inflammation promotes the progress of cancer and CAD. NLR may be a potential biomarker for the association between cancer and CAD.

The results of this study suggest that in addition to common risk factors, there may be another underlying mechanism contributing to the association between the two diseases, in which inflammation may play an important role. It provides guidance for further studies into the association between these two diseases’ cause-and-effect mechanisms and provides a foundation for further studies into the role of inflammation in this relationship. Because these two diseases are related, physicians are encouraged to screen for the other when a patient receives a diagnosis for one, which is the important significance of the study’s conclusions for clinical work. In addition, improved characterization of the interaction between cancer and CVD could ultimately guide further mechanistic efforts to investigate pathophysiology mediating their interaction. Further investigation of the common underlying pathophysiology of cancer and CAD is warranted and may lead to novel therapeutic and diagnostic strategies to improve the care of this growing patient population.

### Study strengths and limitations

This study demonstrated a significant association between newly diagnosed untreated cancer and the anatomical severity of CAD. Furthermore, it analyzes their relationship in different inflammatory situations, as previous studies did not indicate this.

This study has the following limitations: First, as it is a single-center retrospective cross-sectional study, there may be some bias in enrolled patients, data collection, and analysis. Secondly, due to the lack of data on patients’ drug use, the effects of aspirin, statins, and ACEI on the association between cancer and CAD can not be adjusted. Thirdly, the cancer staging and grading data of the enrolled cancer patients were not complete enough to assess cancer severity at the time of diagnosis. Finally, in the retrospective cross-sectional study, we had enrolled all 347 cancer patients, the sample size was not big enough to investigate the correlation between different types of cancer and CAD. In future studies, we will expand the sample size to analyze the relationship between different types of cancer and CAD.

## Conclusions

Cancer was independently associated with the anatomical severity of CAD, however, the association was more significant among patients with high inflammation than those with low inflammation. It indicated that inflammation might play an essential role in the association between cancer and CAD. The results of the study indicated that screening and management of CAD should be taken seriously in cancer patients, especially those with high inflammation. More studies are needed to further investigate the association between cancer and CAD and the underlying mechanism.

## Data Availability

The datasets used and/or analysed during the present study available from the corresponding author on reasonable request.
